# Does the 2013 GOLD classification improve the ability to predict lung function decline, exacerbations and mortality: a post-hoc analysis of the 4-year UPLIFT trial

**DOI:** 10.1186/1471-2466-14-163

**Published:** 2014-10-18

**Authors:** Lucas M A Goossens, Inge Leimer, Norbert Metzdorf, Karin Becker, Maureen PM H Rutten-van Mölken

**Affiliations:** Institute for Medical Technology Assessment, Erasmus University, P.O. Box 1738, 3000 Rotterdam, DR The Netherlands; Boehringer Ingelheim GmbH, Binger Str. 173, 55216 Ingelheim, Germany

**Keywords:** COPD, GOLD classification 2007, GOLD classification 2013, Exacerbations, Lung function decline, Mortality

## Abstract

**Background:**

The 2013 GOLD classification system for COPD distinguishes four stages: A (low symptoms, low exacerbation risk), B (high symptoms, low risk), C (low symptoms, high risk) and D (high symptoms, high risk). Assessment of risk is based on exacerbation history and airflow obstruction, whatever results in a higher risk grouping. The previous system was solely based on airflow obstruction. Earlier studies compared the predictive performance of new and old classification systems with regards to mortality and exacerbations. The objective of this study was to compare the ability of both classifications to predict the number of future (total and severe) exacerbations and mortality in a different patient population, and to add an outcome measure to the comparison: lung function decline.

**Methods:**

Patient-level data from the UPLIFT trial were used to analyze 4-year survival in a Weibull model, with GOLD stages at baseline as covariates. A generalized linear model was used to compare the numbers of exacerbations (total and severe) per stage. Analyses were repeated with stages C and D divided into substages depending on lung function and exacerbation history. Lung function decline was analysed in a repeated measures model.

**Results:**

Mortality increased from A to D, but there was no difference between B and C. For the previous GOLD stages 2–4, survival curves were clearly separated. Yearly exacerbation rates were: 0.53, 0.72 and 0.80 for stages 2–4; and 0.35, 0.45, 0.58 and 0.74 for A-D. Annual rates of lung function decline were: 47, 38 and 26 ml for stages 2–4 and 44, 48, 38 and 39 for stages A-D. With regards to model fit, the new system performed worse at predicting mortality and lung function decline, and better at predicting exacerbations. Distinguishing between the sub-stages of high-risk led to substantial improvements.

**Conclusions:**

The new classification system is a modest step towards a phenotype approach. It is probably an improvement for the prediction of exacerbations, but a deterioration for predicting mortality and lung function decline.

**Trial registration:**

ClinicalTrials.gov NCT00144339 (September 2, 2005).

**Electronic supplementary material:**

The online version of this article (doi:10.1186/1471-2466-14-163) contains supplementary material, which is available to authorized users.

## Background

The Global initiative for chronic Obstructive Lung Disease (GOLD) classification for severity of chronic obstructive pulmonary disease (COPD) is used to classify individual patients, describe study populations, monitor disease progression, and guide individual treatment decisions.

Consensus has grown that the previous GOLD classification, which was entirely based on forced expiratory volume in 1 second as percentage of the predicted value for someone of the same gender, age and height (FEV_1_%-predicted), was an insufficiently reliable predictor of the variety of manifestations of the disease [1–4]. For example, frequent exacerbators are also found among patients with relatively mild forms of airway obstruction [5]. This is important, since exacerbations do not only predict future exacerbations but are also a risk factor of faster disease progression and mortality [6]. There have been pleas for a more explicit recognition of the variety of COPD phenotypes which should improve understanding of the impact of the disease and, more importantly, provide prognostic information and guide the selection of more appropriate therapies [7].

In 2011, GOLD presented a new classification system, which was adapted slightly in 2013 [8]. This new classification distinguishes four groups of patients, based on symptoms and exacerbation risk. The assessment of the latter can be based on either exacerbation history or degree of airflow limitation, whatever results in a higher risk. Symptoms are to be assessed using either the modified British Medical Research Council questionnaire (mMRC) [9], which measures breathlessness, or the COPD Assessment Test (CAT) [10], which provides a more comprehensive assessment of the symptomatic impact of COPD.

Recently, several studies investigated the prognostic value of the old and the new (2011/2013) systems with regards to a number of outcome measures. Mortality was predicted equally well by both systems in studies by Soriano et al., Agustí et al. and Johannessen et al. [11–13], whereas Leivseth et al. found that the old classification performed better [14]. Exacerbations and hospitalisations were predicted better by the new system according to Lange et al. and Agustí et al. [12, 15], but Johannessen et al. saw no difference in performance between the systems [13]. So far, only one study examined the ability of the new system to predict lung function decline [12]. It did not find differences in predicted lung function decline across severity stages. However, in this study no comparison with the old system was made.

It is important that results from these studies are replicated or contradicted in different populations.

Data from the “Understanding Potential Long-term Impacts on Function with Tiotropium” (UPLIFT) trial [16–18] provide the opportunity to investigate the prognostic performance of the new classification system with four years of follow-up. This trial is especially suitable for this purpose, not only because of its duration, but also because of its size (almost 6,000 patients randomized), international origin, and high quality-controlled lung function data.

The aim of this study, therefore, was to compare the ability of the old and the new (i.e. 2013) COPD classification to predict future decline in lung function, mortality, the total number of exacerbations and the number of severe exacerbations.

## Methods

### Data

The UPLIFT trial was a multinational, randomized, double-blind, placebo-controlled trial, investigating the effect of tiotropium on the yearly rate of decline in FEV_1_ in ≥40 years old, currently or formerly smoking patients (≥10 pack-years) with moderate to very severe COPD according to the old GOLD classification system (stages 2 to 4, post-bronchodilator FEV_1_ of 70% or less of the predicted value) [16, 17]. Key exclusion criteria were a history of asthma, a COPD exacerbation or respiratory infection within 4 weeks before screening, a pulmonary resection, use of supplemental oxygen for more than 12 hours per day, and coexisting illnesses that could preclude participation in the study or interfere with the study results.

Patients received either 18 μg of tiotropium or a matching placebo once daily. All respiratory medications, except other inhaled anticholinergic drugs, were permitted during the trial. Smoking cessation programs were offered to all patients before randomization.

Patients were recruited from 2003 to 2004 at 487 centres in 37 countries. The study protocol was approved by the ethics committee at each centre, and all patients provided written informed consent [17]. The follow-up period was four years, in which lung function, exacerbations, St. Georges Respiratory Questionnaire (SGRQ) [19] and mortality were recorded. Exacerbations were defined as an increase in or the new onset of more than one respiratory symptom (cough, sputum, sputum purulence, wheezing, or dyspnoea) lasting three days or more and requiring treatment with an antibiotic or a systemic corticosteroid, and/or a hospitalisation. Patients were assessed at randomisation, after one month, six months, and every six months thereafter. For the base case analyses the data from the two treatment groups (tiotropium and control) were combined.

Data from 5630 patients were used in the analysis.

### Mortality

Time to death was analysed in Weibull regression models, with either the old or new GOLD classification as covariates as well as other prognostic factors. These were selected in an iterative backward selection process, in which the covariate with the highest p-value was excluded until all p-values were below 0.20. Candidates for inclusion in the model were age, gender, body mass index (BMI), smoking status and the presence or absence of several co-morbidities (coronary heart disease, arrhythmia, vascular disease, nervous disease, diabetes, depression and anaemia).

The regression results were used to construct average adjusted survival curves, following the procedure proposed by Hernàn [20]. First, the model coefficients were used to fit multiple individual survival curves for each patient. Each curve assumed a different GOLD stage, irrespective of the actual classification of the patient. The other baseline characteristics were kept constant within patients. After this, mean survival probabilities per 6-month interval were calculated over all patients for each stage and each point in time. These probabilities were then used for constructing survival curves per stage. This was done to assure that differences in the curves would be due to different severity stage assignments only, and not to other differences (e.g. demographic differences) between the groups.

The models’ performance was compared by visually inspecting the ranges over which 4-year mortality differed across stages, by using the Akaike Information Criterion (AIC) for model fit [21] and by Harrell’s c-statistic for the measure of discrimination across stages [22, 23]. A c-statistic of 0.5 means that a model has no predictive discrimination, in other words, that it has a 50% chance of correctly predicting which of two subjects in different risk categories has the highest probability of experiencing the event. There is no universally used interpretation of the value of the c-statistic. In the context of logistic regression, Hosmer et al. consider values of 0.7 to 0.8 to indicate acceptable discrimination, while discrimination is considered excellent between 0.8 and 0.9 and outstanding when the c-statistic ≥0.9 [24].

The AIC is a measure to compare the goodness-of-fit of different statistical models. Its absolute value has no interpretation. A difference in AIC of ≥4 is often considered an indication that the model with the higher AIC fits the data less well [25].

### Exacerbations

Negative binomial regression with adjustment for treatment exposure was applied to analyse the total rate of exacerbations. The regression model contained either the old or the new GOLD stages, as well as other prognostic factors if necessary. In an iterative backward selection process, the covariate with the highest p-value was excluded from the model unless this led to a 10% change in the estimate of the annual exacerbation rate [26].

The regression results were used to estimate mean rates per GOLD stage. For each patient, the number of exacerbations per year was predicted for each stage, given the patient’s characteristics but irrespective of the actual classification of the patient, and assuming 365.25 days per year. The individual predictions per disease stage were then averaged over all patients.

The performance of the new model was compared with that of the old model by visually inspecting the ranges over which rates differed across stages and by using the AIC for model fit. This was repeated for severe exacerbations, which were defined as COPD exacerbations requiring a hospital admission.

### Lung function decline

Lung function decline, expressed as the deteriorating course of post-bronchodilator FEV_1_, was analysed in a linear random effects model. This analysis started at day 30 in order to take into account the fact that many patients experienced an initial post-randomisation improvement in lung function. Covariates were days since randomisation and interactions of GOLD stage and days. These interactions were used to describe decline for each stage. The intercepts and the slope for time since randomisation were assumed to be random with an unstructured covariance matrix and the interactions were modelled as fixed effects. Patients with at least three measurements from day 30 were included. The regression results were used to estimate mean annual lung function decline per GOLD stage. The annual rate of decline per disease stage was determined by multiplying the regression coefficient for this stage by 365.25. The selection of covariates took place along the same lines as for exacerbations. The models’ performance was compared by visually inspecting the ranges over which rates differed across severities and by using the AIC for model fit.

### Classification

Patients were classified into GOLD stage 2 to 4, based on post-bronchodilator FEV_1_% predicted (50-70%, 30-50%, <30%) and into GOLD stage A to D, based on the 2013 GOLD classification [8]. Patients were considered a high risk for an exacerbation if they had a FEV_1_% predicted <50%, or experienced at least two exacerbations in the previous year, or had been admitted to the hospital with an exacerbation at least once during the previous year. The number of exacerbations in the year before randomization was defined as the number of courses of oral corticosteroids or antibiotics or the number of hospitalisations, whichever was the highest.

Since the dataset did not contain CAT or mMRC scores, on which the symptom dimension of the classification is supposed to be based, the Saint Georges Respiratory Questionnaire score (SGRQ) was used instead. The SGRQ measures perceived well-being in COPD patients and the impact of the disease on their activities. Patients with an SGRQ score ≥25 were placed in the ‘high level’ symptoms category. This threshold value was found by Han et al. to have the strongest correspondence with the CAT threshold ≥10 [27].

### Substages

All analyses with the new GOLD classification were repeated with substages of C and D. Patients were assigned to substages based on the reason for being considered high-risk: FEV_1_% predicted <50% but no history of frequent exacerbations (C1 and D1), history of frequent exacerbations but FEV_1_% predicted ≥50% (C2 en D2), or FEV_1_% predicted <50% combined with a history of frequent exacerbations (C3 en D3).

All analyses were performed in Stata 12.1 [28]. Confidence intervals were calculated by bootstrapping with 1000 replications [29, 30].

### Sensitivity analyses

All analyses were repeated with a different threshold for symptom severity: SGRQ ≥39. This value was found by Han et al. to have the strongest correspondence with the mMRC threshold of 2 [27].

Furthermore, the analyses with the SGRQ ≥25 threshold were repeated in the control group separately.

## Results

### Patient characteristics

Table 1 describes the distribution of the patients across old and new GOLD stages. Patients from stage 2 were classified into all four new stages, with the majority in B. Almost all stage 3 and stage 4 patients were classified into stage D.

**Table 1:** **Distribution of patients from stages 2-4 into stages A-D and substages C1-D3**

	GOLD 2	GOLD 3	GOLD 4
	n = 2611 (46% of total)	n = 2529 (45% of total)	n = 490 (9% of total)
A	356 (14% of GOLD2)	-	-
B	1421 (54%)	-	-
C	89 (3%)	195 (8%)	12 (2%)
D	745 (29%)	2334 (92%)	478 (98%)
*C1* ^*^	*-*	*147 (6%)*	*6 (1%)*
*C2* ^*^	*89 (3%)*	*-*	*-*
*C3* ^*^	*-*	*48 (2%)*	*6 (1%)*
*D1* ^*^		*1317 (52%)*	*246 (50%)*
*D2* ^*^	*745 (15%)*		*-*
*D3* ^*^		*1017 (40%)*	*232 (47%)*

^*^ *C1/D1, high risk is based on FEV1<50% only; C2/D2, based on history of frequent exacerbations only; C3/D3, based on both.*

The baseline characteristics of the patients, divided by the new GOLD stages are presented in Table 2. The largest group is formed by patients in stage D. GOLD B contained the highest proportion of current smokers. The time since diagnosis was the longest for D. Airway obstruction was similar for A and B, and for C and D. The number of different types of respiratory medications and the number of courses of antibiotics and oral steroids in the year before randomisation increased from A through D. Patients in A and B had not been admitted to the hospital in the year before randomisation.

**Table 2:** **Baseline characteristics of patients in GOLD stages**

	GOLD A	GOLD B	GOLD C	GOLD D	P-value ^*^
	(n = 356, 6%)	(n = 1421, 25%)	(n = 296, 5%)	(n = 3557, 63%)	
Age	64.9	64.6	64.6	64.5	0.796
Male	81.4%	72.7%	85.8%	73.2%	<0.001
BMI	25.9	26.9	25.7	25.7	<0.001
Current smoker	29.2%	34.3%	24.0%	28.5%	<0.001
Pack-years	37.7	40.4	37.8	40.7	0.076
Time since diagnosis (years)	9.2	9.4	9.2	10.0	0.030
FEV_1_ (liters)	1.72	1.63	1.33	1.16	-
FEV_1_ (as a% of predicted)	60.3%	58.6%	46.5%	41.9%	-
SGRQ	17.1	44.9	18.4	51.3	-
Respiratory medication at baseline (%)					
Short-acting anticholinergic	28.4%	39.4%	40.2%	50.3%	<0.001
Long-acting anticholinergic	2.5%	1.6%	1.7%	2.0%	0.664
Short-acting β_2_-agonist	55.1%	62.5%	60.5%	73.9%	<0.001
Long-acting β_2_-agonist	44.9%	52.2%	58.8%	64.1%	<0.001
Inhaled corticosteroid	47.8%	54.7%	64.5%	66.0%	<0.001
Oral corticosteroid	2.5%	4.6%	4.7%	11.0%	<0.001
Theophylline compound	14.6%	21.0%	23.6%	31.9%	<0.001
Mucolytic agent	3.1%	5.6%	4.7%	8.3%	<0.001
Leuktriene-receptor antagonist	1.1%	2.0%	1.4%	4.3%	<0.001
Supplemental oxygen	0%	0.6%	1.4%	3.0%	<0.001
Number of different types of medication	1.75	2.04	2.24	2.52	<0.001
Number of co-morbidities	3.32	3.71	3.03	3.71	0.588
Number of courses of antibiotics	0.21	0.28	1.08	1.43	-
Number of courses of oral corticosteroids	0.097	0.146	0.55	0.91	-
Number of hospital admissions in previous year	0	0	0.21	0.37	-

^*^Differences were tested using anova or χ^2^ test.

### Mortality

**Figure 1:**
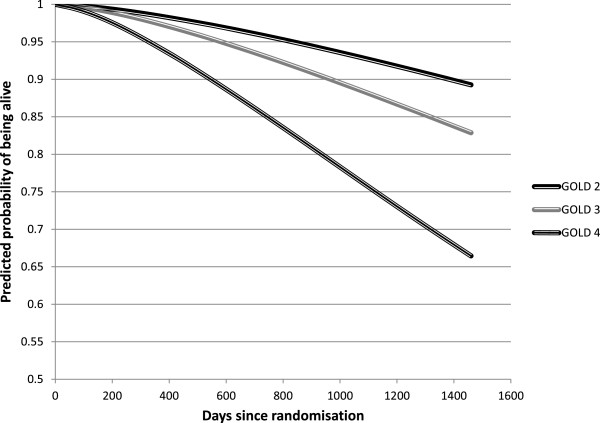
**Model-based adjusted survival curves, per GOLD stage 2, 3 and 4.**

**Figure 2:**
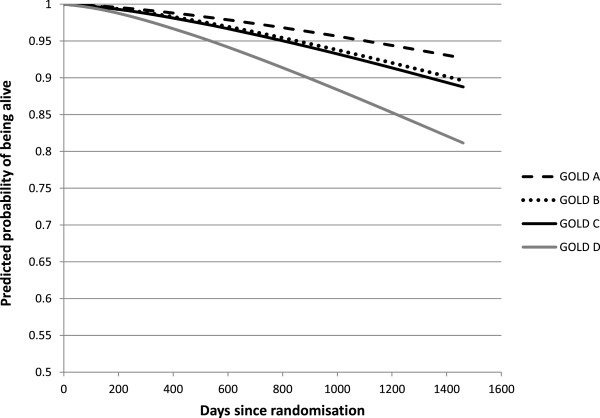
**Model-based adjusted survival curves, per GOLD stage A, B, D and D.**

The covariates in the final model were age, sex, BMI, smoking status and the presence of the comorbidities coronary heart disease, vascular disease, diabetes and depression. The adjusted survival curves for each GOLD stage, adjusted for age, sex and selected co-morbidities are presented in Figures 1 and 2, for the old and the new classification, respectively.

Mortality increases with stage in both systems. However, all differences between stages were statistically significant at the 1%-level for the old classification, while in the new system only the difference between C and D was significant. The curves for B and C almost overlap (p = 0.67). Their distances to stage A are borderline significant (p = 0.08). After 4 years, 7.4% of patients in GOLD A had died, compared to 18.8% in GOLD D. These proportions were further apart for the old stages 2 and 4: 10.7% and 33.5%, respectively.

**Figure 3:**
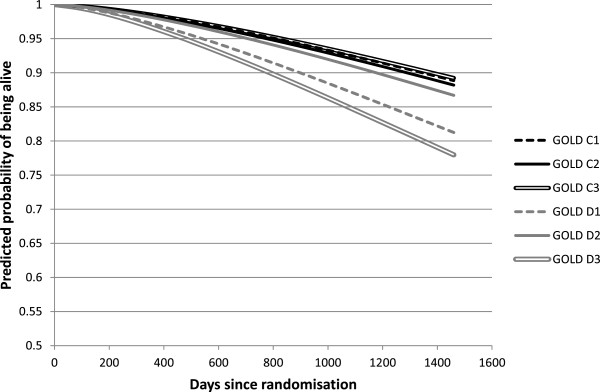
**Model-based adjusted survival curves, per substage of GOLD C and D.**

Table 3 shows that Harrell’s c-statistics for discriminative performance were similar for all three classification systems. All models had a discrimination that falls slightly short of being acceptable, in the interpretation of Hosmer et al. [24]. The best model fit, as measured by the AIC, was achieved for the old model. This was true in over 99% of the bootstrap replications.Figure 3 shows that there were important differences in predicted mortality across substages of D. Patients in substages with a strongly diminished lung function (D1 and D3) were more likely to die than those in D2 (p < 0.01). Being at high risk for exacerbations added relatively little to the mortality risk. In the substages of C, no difference in mortality was found.

**Table 3:** **Weibull models for mortality**

Classification system	C-statistic	AIC
GOLD stage 2 to 4	0.6936	5644.299
GOLD stage A to D	0.6755	5709.177
GOLD stage A, B, C1 to C3 and D1 to D3	0.6861	5693.128

Harrell’s c-statistic and Akaike’s Information Criterion.

### Exacerbations

The final regression models for the total number of exacerbations and for the number of severe exacerbations contained GOLD classification as the sole covariate.

Table 4 shows the annual exacerbation rates for patients classified into the GOLD groups. The exacerbation rate increased with disease severity in both the old and the new system. The rates in the new classification covered a broader range than the rates in the old stages and the new classification system had a much better AIC than the old system.

**Table 4:** **Annual rate of exacerbations (95% confidence interval), per GOLD**

Old stage		P-value ^*^	New stage		P-value ^*^	Substage		P-value ^*^
2	0.53 (0.50 - 0.55)	-	A	0.35 (0.29 - 0.41)	-	C1	0.47 (0.39 - 0.57)	-
3	0.72 (0.69 - 0.76)	<0.001	B	0.45 (0.42 - 0.48)	0.001	C2	0.55 (0.41-0.71)	0.37
4	0.80 (0.72 - 0.89)	0.09	C	0.58 (0.51 - 0.68)	0.001	C3	0.96 (0.70-1.24)	0.06
			D	0.74 (0.71 - 0.77)	0.001	D1	0.56 (0.53-0.60)	-
						D2	0.75 (0.69-0.81)	<0.001
						D3	0.97 (0.92-1.04)	<0.001
AIC	22,697.09			22,571.27			22,417.35	

^*^Wald test of difference with category immediately above. Overall Wald test of equal rates across stages: p < 0.001 for all models. C1/D1: classified in C/D because of lung function impairment; C2/D2: in C/D because of exacerbation history; C3/D3: because of lung function and exacerbation history.

The exacerbation rates varied widely between the substages of C and D. While the exacerbation rate in C1 (no history of frequent exacerbations) was similar to the rate in B, patients in C3 (low lung function and history of frequent exacerbations) experienced more exacerbations than patients in D overall. Symptoms, lung function and exacerbation history were all related to the exacerbation rates.

The patterns are less clear for severe exacerbations (Table 5). The old stages showed a broader range of rates than the new stages. Substages C3 and D3 had the highest number of severe exacerbations, although C3 did not differ from D overall.

**Table 5:** **Annual rate of severe exacerbations (95% confidence interval), per GOLD stage**

Old stage		P-value ^*^	New stage		P-value ^*^	Substage		P-value ^*^
2	0.18 (0.16 - 0.20)	-	A	0.08 (0.05 – 0.12)	-	C1	0.18 (0.12 – 0.25)	-
3	0.39 (0.36 - 0.42)	<0.001	B	0.14 (0.12 – 0.17)	<0.001	C2	0.17 (0.08 – 0.28)	0.71
4	0.54 (0.46 - 0.62)	<0.001	C	0.21 (0.16 – 0.27)	<0.001	C3	0.37 (0.18 – 0.57)	0.001
			D	0.40 (0.37 – 0.43)	<0.001	D1	0.30 (0.27 – 0.33)	-
						D2	0.30 (0.26 – 0.36)	0.85
						D3	0.59 (0.53 – 0.65)	<0.001
AIC	16,019.57			15,859.22			15,634.93	

^*^Wald test of difference with category immediately above. Overall Wald test of equal rates across stages: p < 0.001 for all models. C1/D1: classified in C/D because of lung function impairment; C2/D2: in C/D because of exacerbation history; C3/D3: because of lung function and exacerbation history.

### Lung function decline

The final regression models contained disease severity as the sole covariate. Overall, stages with relatively good lung function at baseline showed a faster decline over the course of the trial (see Table 6). The predicted annual rates of decline covered a broader range for the model with the old stages 2, 3 and 4 than for the model with stages A, B, C and D. Furthermore, the model with the old classification had the best fit in terms of the AIC. The models with the new GOLD classification with and without the substages had a similar fit. The substage of patients who started the trial with a relatively good lung function, C2 and D2, experienced a decline that was comparable to stages A and B. The other substages had stronger declines.

**Table 6:** **Annual rates of lung function decline in millilitres, (95% confidence intervals), per GOLD stage**

Old stage		P-value ^*^	New stage		P-value ^*^	Substage		P-value ^*^
2	47 (44–50)	-	A	44 (38–52)	-	C1	32 (23–42)	-
3	38 (36–41)	<0.001	B	48 (45–52)	0.29	C2	47 (33–60)	0.11
4	26 (21–31)	0.002	C	38 (30–45)	0.01	C3	38 (16–53)	0.43
			D	39 (37–42)	0.74	D1	36 (33–39)	-
						D2	46 (40–52)	0.003
						D3	38 (34–42)	0.04
AIC	-30,229.05			-30,209.08			-30,212.52	

^*^Wald test of difference with category immediately above. Overall Wald test of equal decline across stages: p < 0.001 for all models. C1/D1: classified in C/D because of lung function impairment; C2/D2: in C/D because of exacerbation history; C3/D3: because of lung function and exacerbation history.

### Sensitivity analyses

The results of the sensitivity analyses are presented in the Additional file 1. The same patterns in relative predictive power can be seen as in the base case analyses. Using the SGRQ ≥ 39 threshold, however, led to improvement of all AICs for the new classification system.

Similarly as to what was found in the base case analysis, all three mortality models had very similar c-statistics (Additional file 1: Table S1). In contrast with the primary analysis, the best AIC was achieved by the new mortality model with substages. The old and new classification models had the same fit.

The predicted exacerbation rates in the new classification covered a broader range than the rates in the old classification (Additional file 1: Tables S2 and S3). The new classification system also had a much better AIC than the old system, and the AIC for the new classification with substages was even better. Predicted exacerbation rates were slightly higher when the SGRQ ≥ 39 threshold was used.

With regard to lung function (Additional file 1: Table S4), annual decline rates covered a broader range for the old model, which also had the best AIC. Rates of lung function decline were not different for different SGRQ thresholds.

When the analyses with the original threshold were repeated on the control group separately, similar patterns were found (see Additional file 1: Table S5). For severe exacerbations, the best fit was achieved by models with the new system with the new classification system with substages. For mortality and lung function the best fit was achieved by models with the old classification system.

## Discussion

This study compared the prognostic performance of the old and new GOLD classifications for COPD regarding mortality, exacerbations and decline in lung function. The findings depend on the outcome measure.

As for mortality, both classification systems discriminated equally well, but the old model performed better in terms of model fit. The loss of information on lung function, which was grouped into fewer categories in the new system, does not appear to have been completely mitigated by the added information on symptoms and exacerbation history in the new system.

With regard to (severe) exacerbations, all three dimensions of the new GOLD classification strongly contributed to the predictions. This led to a much better performance for the new classification system than for the old system.

With regard to lung function decline, however, the predictive power of the old system was much better. Information on symptom level and exacerbation history did not improve the ability to predict decline of FEV_1_.

Our study is the first to compare the old and new system’s ability to predict lung function decline. Agusti et al. did assess the decline across the new stages [12] but did not compare the two classification systems. Furthermore, they did not find significant differences in decline, whereas patients with a worse lung function in our data showed a slower decline. This pattern was less clear in the new system than in old system, but still clear and statistically significant. Combining patients with a low lung function and history of frequent exacerbations into the same stages hides the major differences between these patients. This was also observed in earlier studies [12, 15]. Dividing the stages into substages, depending on the reason for which patients are considered high-risk, is very informative and could improve recommendations in individual treatment decisions and in the preparation of treatment guidelines.

The aim of the new guidelines is to enhance the understanding of the impact of COPD on individual patients by combining ‘the symptomatic assessment with the patient’s spirometric classification and/or risk of exacerbations’ [8]. Although lung function in itself does not have a direct impact on patients – it only does so through symptoms, exacerbation risk and mortality risk – it still is an important aspect of disease severity, and hence of the new classification system, because it is a better predictor of mortality than symptoms and exacerbations.

Using trial data for a study like this has advantages and disadvantages. Among the advantages is the high quality of the spirometry data because of there was a good quality control system in place. A disadvantage is that a trial population shows less variation in patient and disease characteristics than a real-life population because of the in- and exclusion criteria. Furthermore, the exacerbation rate in the UPLIFT trial was relatively low. Despite this we found that the new classification system was clearly better in predicting exacerbations than the old classification system.

For all analyses we combined the data from the two treatment groups in the UPLIFT trial. We performed additional analyses with treatment as a covariate. This did not lead to different conclusions.

A limitation of this study is that our data contained no information on the mMRC or CAT scores, which are the recommended ways of establishing symptom severity in COPD patients. However, SGRQ and CAT are highly correlated [31]. According to the authors of the new GOLD guidelines, ‘the crucial aspect is to consider whether the patient has only trivial symptoms or feels significantly limited by them’ [32]. Several scales can be used for that purpose. In fact, the authors note that updates of the guidelines may include other scales.

Nevertheless, different scales may lead to different categorisations. The currently proposed cut-off points of the CAT and mMRC do not lead to exactly the same classification of patients [27, 33]. More specifically, patients were 25% less likely to be classified as C instead of D when the mMRC criterion was applied [27]. The current CAT cut-off point of 10 or more appears to be more in line with a mMRC score of 1 instead of 2 [27, 34].

Earlier studies based the categorisation on the mMRC ≥2 [11–15]. Overall, their findings are in line with ours, using SGRQ ≥25 as a surrogate for CAT ≥10. Furthermore, we found similar results when we used a higher SGRQ threshold as a surrogate for mMRC ≥2 in the comparison of the old and new classification. This is consistent with the guideline statement that does not attach particular importance to the choice for a specific symptom scale. Nevertheless, the model fit was better when the higher SGRQ threshold was used.

In summary, in the UPLIFT population of moderate to very severe COPD patients, the 2013 GOLD classification performed better than the old classification when predicting future exacerbations, whereas the old classification system performed equally well or better when predicting mortality and lung function decline.

## Conclusion

Combining our results in the UPLIFT data with those from earlier studies in different patient populations leads to the conclusion that the new classification system is a modest step towards a phenotype approach. The new system is probably an improvement for the prediction of exacerbations, but a step back with regards to predicting mortality and lung function decline.

## Electronic supplementary material

Additional file 1: Table S1: Weibull models for mortality. **Table S2.** Annual rate of exacerbations, per GOLD (symptom threshold SGRQ ≥39). **Table S3.** Annual rate of severe exacerbations, per GOLD stage (symptom threshold SGRQ ≥39). **Table S4.** Annual rates of lung function decline in millilitres), per GOLD stage (symptom threshold SGRQ ≥39). **Table S5.** AIC scores from analyses in control group. (DOC 58 KB)

## Abbreviations

AIC: Akaike information criterion
BMI: Body mass index
CAT: COPD assessment test (CAT)
COPD: Chronic obstructive pulmonary disease
GOLD: Global initiative for chronic obstructive lung disease
mMRC: Modified British medical research council questionnaire
SGRQ: St. Georges respiratory questionnaire (SGRQ)
UPLIFT: Understanding potential long-term impacts on function with tiotropium.
