# Supplementary material for: Does the 2013 GOLD classification improve the ability to predict lung function decline, exacerbations and mortality: a post-hoc analysis of the 4-year UPLIFT trial
Source: BMC Pulm Med. 2014 Oct 18;14:163. doi: 10.1186/1471-2466-14-163 (PMC4223746; doi:10.1186/1471-2466-14-163)
Supplement: Supplementary file 1 — Additional file 1: Table S1: Weibull models for mortality. Table S2. Annual rate of exacerbations, per GOLD (symptom threshold SGRQ ≥39). Table S3. Annual rate of severe exacerbations, per GOLD stage (symptom threshold SGRQ ≥39). Table S4. Annual rates of lung function decline in millilitres), per GOLD stage (symptom threshold SGRQ ≥39). Table S5. AIC scores from analyses in control group. (DOC 58 KB) [file 12890_2014_601_MOESM1_ESM.doc]

# Additional file 1

***Table S1. Weibull models for mortality. Harrell’s c-statistic and Akaike’s Information Criterion (symptom threshold SGRQ* ≥*39)***

| **Classification system** | **C-statistic** | **AIC** |
| --- | --- | --- |
| GOLD stage 2 to 4 | 0.6936 | 5644.299 |
| GOLD stage A to D | 0.6900 | 5644.952 |
| GOLD stage A, B, C1 to D3 | 0.6928 | 5635.210 |

***Table S2. Annual rate of exacerbations, per GOLD (symptom threshold SGRQ* ≥*39)***

| **Old stage** | | **New stage** | | **Substage** | |
| --- | --- | --- | --- | --- | --- |
| 2 | 0.53 | A | 0.41 | C1 | 0.48 |
| 3 | 0.72 | B | 0.45 | C2 | 0.59 |
| 4 | 0.80 | C | 0.59 | C3 | 0.83 |
|  |  | D | 0.79 | D1 | 0.60 |
|  |  |  |  | D2 | 0.80 |
|  |  |  |  | D3 | 1.01 |
| AIC | 22,697.09 |  | 22,541.49 |  | 22,404.15 |

Overall Wald test of equal rates across stages: p<0.001 for all models. C1/D1: classified in C/D because of lung function impairment; C2/D2: in C/D because of exacerbation history; C3/D3: because of lung function and exacerbation history.

***Table S3. Annual rate of severe exacerbations, per GOLD stage (symptom threshold* SGRQ ≥39)**

| **Old stage** | | **New stage** | | **Substage** | |
| --- | --- | --- | --- | --- | --- |
| 2 | 0.18 | A | 0.11 | C1 | 0.22 |
| 3 | 0.39 | B | 0.15 | C2 | 0.19 |
| 4 | 0.54 | C | 0.26 | C3 | 0.43 |
|  |  | D | 0.44 | D1 | 0.33 |
|  |  |  |  | D2 | 0.34 |
|  |  |  |  | D3 | 0.62 |
| AIC | 16,019.57 |  | 15,808.99 |  | 15,604.74 |

Overall Wald test.of equal rates across stages: p<0.001 for all models. C1/D1: classified in C/D because of lung function impairment; C2/D2: in C/D because of exacerbation history; C3/D3: because of lung function and exacerbation history.

***Table S4. Annual rates of lung function decline in millilitres), per GOLD stage (symptom threshold SGRQ* ≥*39)***

| **Old stage** | | **New stage** | | **Substage** | |
| --- | --- | --- | --- | --- | --- |
| 2 | 47 (44–50) | A | 44 | C1 | 37 (23–42) |
| 3 | 38 (36–41) | B | 51 | C2 | 42 (33–60) |
| 4 | 26 (21–31) | C | 38 | C3 | 36 (16–53) |
|  |  | D | 39 | D1 | 35 (33–39) |
|  |  |  |  | D2 | 47 (40–52) |
|  |  |  |  | D3 | 38 (34–42) |
| AIC | −30,229.05 |  | −30,214.41 |  | −30,219.20 |

Overall Wald test of equal decline across stages: p<0.001 for all models. C1/D1: classified in C/D because of lung function impairment; C2/D2: in C/D because of exacerbation history; C3/D3: because of lung function and exacerbation history.

***Table S5. AIC scores from analyses in control group***

|  | **Classification system** | | |
| --- | --- | --- | --- |
| **Outcome** | **GOLD stages 2 to 4** | **GOLD stages A to D** | **GOLD stages A, B and C1 to D3** |
| Mortality | 2945.11 | 2984.10 | 2982.28 |
| Exacerbations | 11592.57 | 11553.05 | 11469.95 |
| Severe exacerbations | 8302.08 | 8212.97 | 8081.42 |
| Lung function decline | −14784.91 | −14758.82 | −14768.52 |
